# Experiences With In-Person and Virtual Health Care Services for People With Chronic Obstructive Pulmonary Disease: Qualitative Study

**DOI:** 10.2196/43237

**Published:** 2023-08-14

**Authors:** Thea Krag, Emma Højgaard Jørgensen, Klaus Phanareth, Lars Kayser

**Affiliations:** 1 Department of Public Health University of Copenhagen Copenhagen K Denmark; 2 Epital Health Ltd Søborg Denmark

**Keywords:** chronic obstructive pulmonary disease, telemedicine, telehealth, virtual RCC service, rehabilitation, self-management, eHealth literacy, social support, well-being

## Abstract

**Background:**

The World Health Organization and the European Commission predict increased use of health technologies in the future care for patients in Europe. Studies have shown that services based on telehealth, which includes components of education, as well as rehabilitation initiatives can support the self-management of individuals living with COPD. This raises an interest in how virtual and in-person interactions and roles can best be organized in a way that suits people living with COPD in relation to their treatment and rehabilitation.

**Objective:**

This study aims to investigate how individuals living with COPD experience different combinations of virtual and in-person care, to help us better understand what aspects are valued and how to best combine elements of these services in future care.

**Methods:**

Two rounds of semistructured interviews were conducted with 13 and 4 informants, respectively. The individuals were all recruited in relation to a research project led by the telehealth initiative Epital Health. The first round of interviews included 11 informants, as 2 dropped out. Of these, 7 received the telemedicine service provided by Epital Health, 3 participated in a 12-week COPD program provided by their respective municipality, and 1 did not receive any supplementary service besides the usual care. In the second round, which included 4 informants, all had at one point received the telemedicine service and participated in a municipality-based rehabilitation program. A content analysis of the interviews was performed based on deductive coding with 4 categories, namely, (1) Self-management, (2) Health-related support, (3) Digital context, and (4) Well-being.

**Results:**

Medical and emotional support from health care professionals is a key aspect of care for individuals with COPD. Acute treatment with at-home medicine, monitoring one’s own condition through technology, and having easy access and close contact with health care professionals familiar to them can promote self-management and well-being, as well as provide a feeling of security. Having regular meetings with a network of peers and health care professionals provides education, support, and tools to cope with the condition and improve own health. Furthermore, group-based activity motivates and increases the activity level of the individuals. Continued offers of services are desired as many experience a decrease in achieved benefits after the service ends. More emphasis is placed on the importance of the therapeutic and medical elements of care compared with factors such as technology. The identified barriers related to optimal utilization of the virtual service were related to differentiation in levels of contact depending on disease severity and skills related to the practical use of equipment.

**Conclusions:**

A combination of virtual and in-person services providing lasting medical and social support is suggested for the future. This should build upon the preferences and needs of individuals living with COPD and support relationships to caregivers and peers.

## Introduction

### Background

Chronic obstructive pulmonary disease (COPD) is the third leading cause of death globally with 3.23 million deaths reported in 2019 [1]. The condition is characterized by breathlessness (dyspnea), coughing, increased sputum, and tiredness [1]. This often results in reduced physical activity, sleep disturbances, the experience of social isolation, anxiety, and depression [2-5]. The World Health Organization Regional Office for Europe (WHO/Europe) and the European Commission predict an increase in the integration of telemedicine and health technologies for the treatment of patients in Europe. Following the approval of the Regional Digital Health Action Plan for 2023-2030 by the Ministers of Health at the WHO Regional Committee for Europe in September 2022 [6,7], there is an expectation for a new way of collaborative management between health care professionals (HCPs) and patients. This makes it more relevant than ever to gain insights into what expectations patients have for their treatment and what they value in already experienced treatment programs. This knowledge can contribute to an understanding of what elements of care should be considered when designing and providing new ways of caring for patients with COPD, and how the use of technology can help to alleviate current shortcomings in treatment as experienced today by patients globally.

The Global Initiative for Chronic Obstructive Lung Disease (GOLD) recommends pulmonary rehabilitation as part of integrated patient management [8]. Pulmonary rehabilitation, as defined by an expert group from the American Thoracic Society together with the European Respiratory Society, is “a comprehensive intervention based on thorough patient assessment followed by a patient-tailored therapy that includes, but is not limited to, exercise training, education, self-management intervention aiming at behavior change, designed to improve the physical and psychological condition of people with chronic respiratory disease and to improve the long-term adherence to health-enhancing behaviors” [9].

The GOLD recommends that rehabilitation programs should last from 6 to 8 weeks and include a range of HCPs to cover the different aspects of patient education [8].

The motivation for this study originated from our previous work concerning the development and implementation of a 24/7 telemedicine service for individuals with COPD [10]. In this work we, among others, discuss the extent, as well as the related challenges, to which virtual and in-person services can most efficiently be combined to offer a digitally assisted active and independent living that meets the needs, preferences, and values of people with COPD [10,11]. To better understand this concept, we will build on a recently developed model, the Readiness and Enablement Index for Health Technology (READHY) [12-14], as a theoretical framework that helps us to enlighten aspects of the role of support by peers and professionals, their digital health literacy, and their ability to self-manage. By using this lens in interviews with a group of individuals having an experience that covers a range of combinations of exposures to virtual and in-person medical treatment and rehabilitation services, it is anticipated to help us better understand how to best combine services that are offered virtually or in-person, which may create better support from peers and professionals and increase ease of access to technology, increase self-management, and achieve a higher level of well-being.

### Role of a Supportive Network in COPD

Social support may be a factor for the improvement of self-care behavior, treatment adherence, and self-efficacy among patients with COPD. It may also help maintain or improve their overall functioning (covering 6 domains as defined by the World Health Organization Disability Assessment Schedule 2.0 [WHODAS II] score) [15,16]. Further, a higher degree of social support is associated with lower levels of depression and anxiety, as measured by The Beck Depression Inventory (BDI) and The State-Trait Anxiety Inventory (FormX-2, Trait Anxiety). Likewise, negative social support (eg, an unsympathetic response and the feeling of being let down by social network members) is associated with higher levels of depression and anxiety [17,18]. Anxiety, in turn, is associated with poor health-related outcomes, such as poor physical health status and performance, risk of COPD-specific deterioration and exacerbations, functional limitation, and lower disease-specific quality of life [17-19]. Interventions aimed at increasing social support, fostering self-efficacy, and reducing anxiety can help maintain overall functioning among patients with a chronic condition [16].

### Role of Technology

It is generally believed that digital tools can promote patient empowerment by enhancing the one’s ability to understand and influence their own health status, enabling distant clinical support, and increasing the ability to manage the disease in an at-home setting [11,20]. This is in contrast to our recent study, where we found that participants in a 24/7 accessible virtual response and coordination center (RCC) service felt less active over time in managing their health [21]. This may indicate a decrease in independence, but could also be an indicator of the virtual RCC service easing the management of their health.

With the prospect of increased use of telemedicine and health technologies in the future care for individuals with COPD [6,7], it is important to understand the individual preferences and values in relation to virtual and in-person services for successful planning of future health services. To meet this need for information, we herein report on the problems individuals with COPD experience in their care today, and how this can be managed by virtual and in-person services. The main objective is to identify the elements of the different services explored in this study, namely, a virtual RCC service, in-person municipality-based rehabilitation programs, and no specific initiatives besides regular care, informants value in their care, and those that would be important to maintain in a future care model. To obtain this information an analytical framework inspired by READHY [12] will be adopted with increased mental well-being as a goal.

### Self-management

Self-management has been defined in many ways, but in the context of living with a chronic condition it is referred to as the ability to deal with all that a chronic illness entails, including symptoms, treatment, physical and social consequences, and lifestyle changes [22]. It is essential to improve both general and mental health, which in this context is “a state of well-being in which an individual realizes his or her own abilities, can cope with the normal stresses of life, can work productively, and is able to make a contribution to his or her community” [23]. A review from 2022 reported that interventions to improve self-management in patients with COPD are associated with improved outcomes including improvements in health-related quality of life (measured by the Saint George Respiratory Questionnaire), lower probability of respiratory-related hospital admissions, and no excess respiratory-related and all-cause mortality risks [24]. It may also improve physical activity and performance, as well as emotional function, and reduce the number of emergency room visits [25-28].

Interventions aimed at improving mental health as well as symptom management prove more effective than those solely aimed toward symptom management [27]. This is supported by the WHO, which defines health as “a state of complete physical, mental and social well-being and not merely the absence of disease or infirmity” [23]. Examples of self-management strategies are breathing exercises, physical activity, and techniques to perform daily activities [29].

A Norwegian study from 2018, using the Health Education Impact Questionnaire (HeiQ) to assess the ability of patients with COPD to manage their condition and how this affects their condition, found that a higher symptom burden from COPD was associated with a lower level of self-reported ability to manage their own condition. This was related to t-scores in 6 out of 8 scales [30].

Many patients with COPD have limited knowledge about their condition, including the cause of the disease, the consequences of inadequate therapy, and the management and prevention of exacerbations [31]. Insufficient knowledge is related to poor adherence to medical treatment and as a consequence a lack of experience of its benefits [15].

## Methods

### Study Overview

This study is reported according to the COREQ (Consolidated Criteria for Reporting Qualitative Research) [32] and follows the recommendations by Connelly [33] and Lee [34] to ensure trustworthiness.

### Study Setting and Context

Alles Lægehus is an organization providing primary health care in 17 general practice centers in Denmark.

In Denmark, visits to the general practitioner (GP) are normally free of charge for the patients, as the GPs are reimbursed by 1 of the 5 Danish regions. The GPs are either working alone or organized in joint GP centers, where typically 3-8 physicians work together and are assisted by registered nurses and medical secretaries. In some areas of Denmark, it is difficult to recruit GPs. In these areas, the GP services are offered by medical doctors employed by either the Danish regions or private organizations. They can be former GPs or recruited from hospitals and are employed full-time, part-time, or are temporarily visiting. They can be located in either individual practices or GP centers.

### Study Design and Interview Participants

The informants for this study are recruited among those participating in an ongoing randomized controlled trial, the TEMOCAP study (Telemonitoring of COPD in General Practice), which was initiated in September 2020 by KP and LK. The informants had, in the original informed consent form, allowed being contacted for substudies. The TEMOCAP study is conducted in collaboration with the University of Copenhagen, Epital Health Ltd, and Alles Lægehus. In the TEMOCAP study, 186/200 individuals with COPD were randomized either to usual care (services from GPs) or to a virtual RCC service in addition to their usual care. The RCC service, provided by Epital Health, is based on the principles of the Epital Care Model (ECM), which is a Danish telehealth initiative developed as a cocreative process involving all stakeholders participating in COPD treatment and care [35]. The model is designed to promote integrated people-centered health service and facilitate engagement, self-management, and empowerment of patients, and has been iteratively developed and tested since 2012. The ECM is described in more detail elsewhere [11,21,35]. The current implementation of the ECM has focused on the medical treatment of COPD and less on rehabilitation and services from allied health professionals such as occupational therapists, physical therapists, dietitians, and health coaches, despite all these being part of the conceptual framework of the ECM [10]. When enrolled in Epital Health as part of the TEMOCAP study, the participants are recruited from 1 out of 4 selected GP centers, organized by Alles Lægehus. The 4 centers were selected to ensure a geographical spread in the inclusion of participants, so there is population heterogeneity.

**Figure 1:**
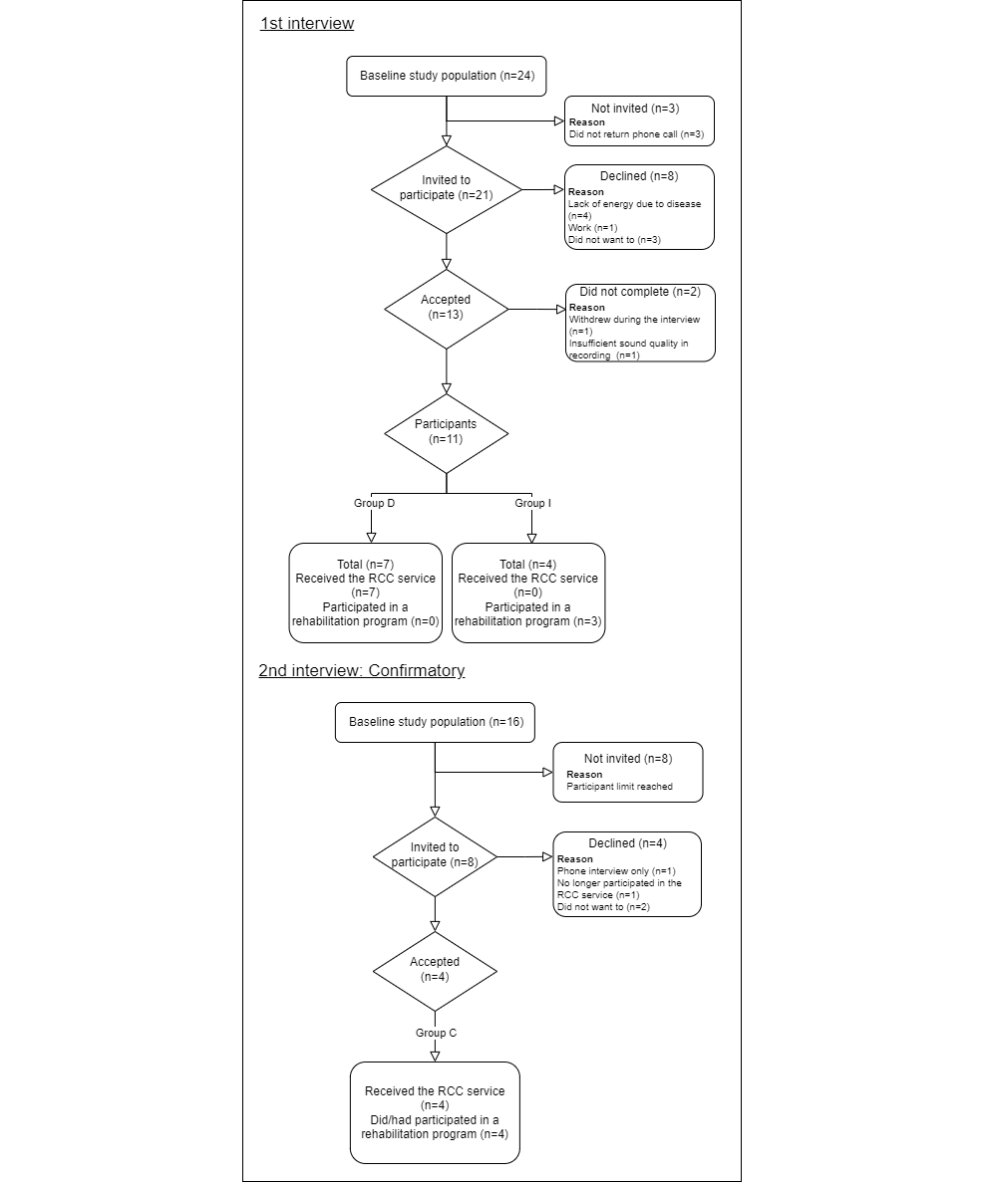
Recruitment process for interview participants.

Via the TEMOCAP study, we were able to recruit 13 informants in April 2021 for the first round of interviews, and 4 informants in November 2021 for an additional round of interviews. Figure 1 illustrates the recruitment process for the interview participants.

Reasons for declining were not requested. However, some mentioned declining participation due to undergoing or having undergone severe illness and therefore not having the capacity to participate.

In total, 24 individuals were invited to this study by the principal investigator of the TEMOCAP study (KP) from the included participants (on the top of the list based on inclusion date) from 2 GP centers. The participants were recruited from 2 different municipalities; 11 females and 13 males (12 in the category of receiving the virtual RCC service and 12 in the category of not receiving the RCC service, respectively) were invited via phone to participate in the first semistructured interview (Multimedia Appendix 1). Of these, 13 (3 females and 10 males) accepted the invitation. However, data from 2 of the interviews were not included for the following reasons: informant did not complete the interview (n=1) and quality of the recording was not sufficient for transcription (n=1). The remaining 11 informants ranged in age from 48 to 81 years; 7 of those who accepted the invitation received the virtual RCC service and 4 did not. The second interview was a confirmatory interview with a focus on the combined experience of physical and virtual services. Here, 4 new informants (age range 46-87 years), 2 females and 2 males, had all received the virtual RCC service and had at one point participated in a rehabilitation program. The purpose of the confirmatory interview was to present the results from the first interview to informants exposed to both virtual and physical services and clarify whether our findings matched their experience (Multimedia Appendix 2). The duration of rehabilitation programs lasted from once a week for 5 weeks to twice a week for 12 weeks.

The interview was conducted virtually because of COVID-19, and to facilitate the inclusion of informants living in various locations. The first interviews took place in April 2021 and the second confirmatory interviews took place in November 2021. The first author (TK, female) and the second author (EHJ, female) performed 9 and 6 of the interviews, respectively. Both authors are finalizing their bachelor’s degree in health informatics with this study at the University of Copenhagen, qualified in performing qualitative and quantitative methods during their study, and for this final project were trained and supervised by author LK, who is experienced in performing qualitative methods and an associate professor at the Department of Public Health, University of Copenhagen. The interviewers followed an interview guide (Multimedia Appendix 1), where the questions were based on themes inspired by the READHY model [12] and the WHO-5 Well-being Index [36-38]. The interview guide was pilot tested before the interviews.

The interviews were performed over Zoom (Zoom Video Communications, Inc.) or via a phone call, as some participants had technical difficulties with using Zoom. Informants joined from home, and the interview was scheduled for a duration of 30 minutes. For some, the interviews lasted for 20 minutes, whereas for others it lasted up to an hour. The duration of the interviews was related to the number and kinds of services the informant had experienced and their ability to express themselves. The informants were not invited to review transcripts or data after data collection.

The education level among the informants ranged from only school education (n=4) to professional training (n=7) and academic training (n=2). For 2 informants, this information was not obtained.

Informants’ statements related to other self-services such as apps or additional self-monitoring equipment acquired will not be included in the reporting of results.

Table 1 presents the components of the 3 services and their correlation with the WHO recommendations. These are based on information from the WHOs global strategy on people-centered and integrated health services [39].

**Table 1:** COPD^a^ service alignment with GOLD^b^-related aims.

Service	Telehealth	Rehabilitation programs (Municipality)	Other (eg, general practitioner, outpatient clinic)
GOLD-related aims	Self-management education, pharmacotherapy, preventing exacerbations	Programs lasting 6-8 weeks, self-management education to improve the physical and psychological condition, active lifestyle and exercise, offer to participate in smoking cessation course, variety of HCPs^c^	Variety of HCPs, regular assessment, influenza and pneumococcal vaccination, pharmacotherapy
Description	24-hour individual service with daily monitoring and assessment of the condition	Group-based rehabilitation course with education and physical activity with attendance 2 times per week	Annual visitation or when needed
Duration	As long as registered	6-8 weeks	Permanent
Components	Self-reported outcome measures	Physical exercise, smoking cessation, nutritional counseling, training in daily activities, and patient education; additionally, network and exchange of knowledge	Annual checks, vaccinations, medication
Personnel	HCPs and staff (response and coordination center staff)	Nurses, nutritionists, and physiotherapists	Doctors and other HCPs

^a^COPD: chronic obstructive pulmonary disease.

^b^GOLD: Global Initiative for Chronic Obstructive Lung Disease.

^a^HCP: health care professional.

### Data Analysis

Interviews and analysis were conducted in Danish, which is the native language of the researchers and informants. In the “Results” section, selected quotes are presented, which were translated by the first author (TK) and verified by authors EHJ and LK. The translated selected quotes are used to illustrate our analysis and arguments.

The interviews were transcribed, read through, coded, and analyzed using deductive content analysis in close collaboration between the 2 interviewers TK and EHJ. They were discussed with LK biweekly [40]. A codebook [41] was constructed with 4 categories based on the 3 themes from READHY (ie, Health-related support, Digital context, and Self-management [12]) and the focus on the informants well-being, as this is a recommended outcome of health defined by the WHO [2]. NVivo 12 (QSR International) was used to organize and code the data based on the codebook. For alignment and insurance of the relevance of codes, the coding of the first interviews was discussed by the authors and the new codes that evolved from the coding and discussion were added to the codebook.

In the “Results” section, we present the results based on the content analysis applied for coding, which were stratified into the following 4 categories: Health-related support, Digital context, Self-management, and Well-being. Health-related support is divided into 2 subcategories: Medical, provided by formal caregivers (HCPs), and social network provided by informal caregivers (spouses, relatives, and friends). Although there can be an overlap between different responsibilities and roles, we chose, for the sake of a later discussion, to make this distinction in the presentation.

We here report on patients’ ability to manage their COPD-related condition, how they feel supported, and to what extent the services impact their well-being.

### Ethical Considerations

The informants were given information about the study, researchers, and the collection and handling of data in accordance with the Helsinki Declaration in both written and oral forms. They were also informed that their participation was voluntary and anonymous and that they at any point could withdraw their consent without changing their role in the randomized controlled trial. Informed consent was obtained before the interview. During the recorded interview, the consent was documented. As no biological material was used in the study, review, approval, or exemption from the Danish National Center for Ethics was not required, according to Danish legislation [42]. Furthermore, local institutional committees do not exist in Denmark. The interviews were performed and recorded over an encrypted version of Zoom licensed by the University of Copenhagen. All data from the informants are considered personal health data and stored on safe drives and handled in accordance with Danish legislation (General Data Protection Regulation). The informants were not compensated for participation and were not invited to review transcripts or data after data collection.

## Results

Table 2 presents the demographics of the informants.

**Table 2:** Characteristics of the informants.

ID	Sex	Age (years)	Years lived with chronic obstructive pulmonary disease	Marital status
D1^a^	Male	81	16	Married
D2	Male	75	15	Married
D3	Female	64	10	Married
D4	Male	70	5-6	Single
D5	Male	72	10-15	Married
D6	Male	69	5-10	Single
D7	Male	63	10	Single
I8^b^	Male	48	7	Single
I9	Female	75	N/A^c^	Single
I10	Male	56	1	In a relationship
I11	Female	69	10	Single
C12^d^	Female	46	5	Single
C13	Male	87	6	Married
C14	Male	65	9 (20 with symptoms)	Single
C15	Female	65	8	Married

^a^D: Was part of the first round of interviews and participated in the virtual response and coordination center service.

^b^I: Was part of the first round of interviews and was not offered the virtual response and coordination center service.

^c^N/A: not applicable.

^d^C: Was part of the second confirmatory interview and participated in the virtual response and coordination center service and attended a chronic obstructive pulmonary disease rehabilitation program.

### Health-Related Support

#### Medical

For many, a medical doctor is more than a professional treating a disease. This was reflected equally in both rounds of the interviews: when dealing with a chronic condition, many wish for the doctor to become a close source of support who not only treats symptoms and prescribes medication. Based on informants’ statements, it emerges that there is a wish for what many would describe as coherent care. More specifically, it is important for individuals with COPD to be treated by GPs who are familiar with their medical history and involved and show interest in their diagnosis-related well-being both medically and emotionally.

Feeling sufficiently medicated and having time to be listened to and understood by the GP is an important aspect of care as described by the informants of this study. When asked about what could be improved in one’s care, one of the informants commented as follows:

That someone would listen to how I feel. Both personally and with the breathing, you know? (...) The only time I felt like I was listened to was by the doctor from the Epital (virtual RCC service) that’s really more than the other clowns (doctors) who don’t bother to listen. They will just say that I should take more and more of this s***** medication. Well, it won’t help to take more, when the medication is not working”.

ID I8

Because of short consultations in general practice, the switch between doctors within GP centers, and the absence of COPD specialists, many experienced an unmet need for such coherence, as explained by the following quotes:

Then I came into something called a GP center, and the times I have now been to the doctor, I have now met 13 different doctors, so I don’t get into close contact with any doctor to talk about this (their condition)

ID I11

You only have 7 minutes with your doctor, right? So if you need to sit and go through your entire medical history each time, then those minutes will quickly pass

ID D3

The desire for such care was emphasized by having experienced a virtual RCC service with continuous monitoring involving RCC staff, which includes HCPs and non-HCPs specialized in COPD. This provided an experience of feeling understood and, in some cases, being better medicated after consultations with the doctor. Those who had a more severe condition were more frequently contacted by the RCC staff, which along with being monitored provided a sense of security. This was emphasized by the following comments:

First of all, it really depends on the doctor you have. Their attitude towards COPD in general ‘well, are you a smoker’? Well then, it’s just the way it is, you have COPD, you just have to live with it. Turns out I also had asthma and pulmonary emphysema (...) meanwhile, at the Epital (virtual RCC service), they know exactly how it is. You don’t have to explain and argument why you think you feel bad, cause they know

ID C12

Then I will get a call every day and they will let me know if I should take some penicillin or prednisolone, which I have laying around as part of the provided acute medicine. So one is really followed closely. That’s nice. It’s also nice to have, so I don’t have to go the doctors first, and then to the pharmacy (...) it makes one feel safe.

ID D3

Correspondingly, those with a less severe condition had less contact with the RCC staff. However, some of these informants expressed a desire to have more contact, although this was expressed with hesitance:

They could call a little more often, you know? (...) I would have liked for him (doctor from the virtual RCC) to call and ask “how are you feeling?” and we could have a chat. But no, this is nonsense, overall, it’s alright

ID D1

The monitoring also appeared to affect medication adherence, as one described becoming more systematic and compliant after being monitored:

I stopped taking my medication because it didn’t work after the first week, so it didn’t really matter you know? Well, then I was enrolled in this (virtual RCC service), and I was forced to be more systematic. And nothing happened in the four first months. But then all of sudden, something started to happen. Eh, quite significantly actually I would say

ID D5

#### Social Network

Having social support from HCPs as well as a social network does, for some, play an important role when dealing with a life-threatening disease. For many, the symptoms of the condition are accompanied by anxiety and new routines in daily life. The interviews indicate that this can be helped by relying on HCPs and peers, who can provide therapeutic and educational support.

While some rely on their social network for these needs (spouse, relatives, or friends), others intentionally choose to use secondary relations, such as HCPs and peers, if available. In this study, the informants experienced or expected to experience such support through their rehabilitation program and from the virtual RCC, as illustrated by the following comments:

I think I get some good inputs there (the rehabilitation program) (...) I feel like I can also feel an improvement. We do some exercises, have some lectures and then some learning afterwards. I am sure that when these 12 weeks are completed, it will have helped me on way or another, you know?

ID I10

I have anxiety about not being able to breathe, or to get suffocated, to put it bluntly. It is very much associated with anxiety for me (...) I am very happy to attend this rehabilitation program because I am confident and hopeful that I will learn how to change my behavior, so I don’t get so much anxiety, really. It’s a bit disabling I think

ID I11

Rehabilitation groups provide the setting to talk to individuals in a similar situation and can give a sense of “being in it together”:

We exchange experiences and ask each other ‘are you experiencing this too?’ - because it is a safe environment (...)

ID C12

Virtual services provide accessibility to well-known RCC staff who can provide support from a professional standpoint. In both cases, informants indicated that having these resources meant opportunities to have conversations and support that could otherwise be difficult to have with social networks due to not wishing to burden or be of concern to their relatives.

>Well, my daughter has an understanding but (...) I think she is a bit afraid (...) I have good support in my daughter also (...) but it’s better to have this network now (the virtual RCC service), they simply understand it better (...) I also have a huge network (in a setup similar to the rehabilitation program in format but not time-limited). I go down there and wine and cry, and laugh and sob if something is bothering me (...) it’s nice to have this network too (rehabilitation program) where we are peers and have the same medical condition [ID I11].

### Digital Context

For many who participated in the virtual RCC service, the usage of technology was an embedded practice that they did not address or were not conscious of in their everyday life. Besides, it was not until the equipment did not perform as expected that it became a nuisance or entered their daily life as a factor to be addressed. When establishing services such as a virtual service with digital equipment, there can be risks of introducing technologies that may be difficult for individuals to use. For example, for the virtual RCC service, some participants abandoned or failed to measure their lung capacity with spirometry twice daily due to breathlessness and the mouthpiece of the equipment not fitting. One of the informants said,

Sometimes when I puff the third time to get it stated... I buff, and then I cough and am about to die. I do two puffs and if it doesn’t take it, then I won’t bother. I just move on to the next

ID D4

This can cause participants of the virtual RCC service to miss the potential benefits or perhaps even abandon the RCC service all together:

The spirometer and I simply didn’t get along, so I ended up spraining a muscle in my chest. So that’s when it stopped (the RCC service).

ID C12

Lack of mental surplus, forgetfulness, tiredness, and having to bring equipment when being away from home for longer periods are also factors that can create noncompliance in providing spirometry data twice daily, as one of the informants said,

Sometimes I work, and I come home late, you know? So sometimes I’ve had to skip it because it’s gotten so late in the evening. If you’re in the city or something else or on vacation, where you have to bring it... I’ve had difficulty installing it in those cases

ID C15

While this became a barrier to the RCC service for some of the informants, others expressed a determination to persevere and deal with the equipment until it worked for them. It appeared to be related to the level of skill:

When I had to change the batteries on this oxygen saturation device. Oh my god, that is advanced. It’s not just something you do. You have to figure out how to take it apart. And when you finally take it apart, you can’t re-assemble it. (...) good one is born with a certain amount of stubbornness. I won’t give up before it works

ID D3

The sharing of personal data did not appear to be a worry among the participants, and the few that spoke of the subject stated that it did not have any significance, as they had trusted that no one would misuse their data or personal information. One informant said,

Why would I have anything against that? (Sharing personal data with the RCC service). I don’t assume that they would... the only thing they could misuse is my social security number (...) I don’t go around being worried about things like that

ID D6

### Self-management

Many of the informants have an interest in acquiring knowledge as well as developing skills and insight that help them manage their condition. Many wish to be less dependent, have fewer hospital visits, receive optimal treatment based on their condition, and have a sense of control and security.

The informants’ statements indicate that factors promoting self-management include access to information, which is relevant and mediated in a tailored and pertinent manner. Many have smartphones, computers, and access to an abundance of information. Despite this access, the ability to utilize the information requires facilitation. Several participants noted that when HCPs and peers provided tailored information and guidance on how to use, for example, equipment, medication, and services in the health care sector, this would make one more likely to take advantage of the information and reflect on new behavior and choices. As one informant stated,

I have been made aware during this program (Rehabilitation), that I can actually use my inhalator in advance... this one that I can take when needed. I wasn’t told that by the doctor who prescribed it (...) at the same time, I have just been summoned to the lung department, for another examination. (...) simply because I was encouraged by one of the others at the program (...) I would like to be sure that I am getting the right medicine, and I hope I can get a clarification on that

ID I10

For some, tools enable monitoring and create an ability to follow the condition and support the ability to act. Having the resources available at home and before the need arises can give the individual the ability to take responsibility as well as use what is relevant to them. For example, participants of the RCC service had access to a box with medicine to be used for acute treatment, which was associated with rapid action upon exacerbations and initiation of treatment both with and without initial consulting with HCPs. This is perceived as something that creates less dependence and prevents visits to the hospital.

I was feeling worse and worse (believed to have pneumonia) (...) I have gotten to that point where I don’t want to go to the doctor when I am feeling so bad. But then it was really nice to be able to start treatment myself – and it worked, clearly

ID C12

Sometimes I have to take a round of it (acute medicine) and then I feel better – and that means I don’t have to go to the hospital every time I am losing my breath, they can fix it just by giving me some of the medication I have laying around at home

ID D7

Having the ability to monitor own condition through equipment and interpreting data can help create meaning between the current state and medication dynamics. By contrast, enabling the RCC staff to monitor an individual’s current state might result in less engagement, because the individual can rely on being monitored and thereby be less alert. As an example, some align their concerns with the feedback they receive from their virtual service, where no contact upon sending data is perceived as an indication of a stable condition:

When I send the data, then there is also someone who, kind of like, watches over me

ID D4

And then I can say if I don’t get the call, then it’s not so bad after all

ID D3

There is a difference in how informants may perceive their ability and role in relation to services. One informant reported a coresponsibility based on insights into her condition, which made her actively involved, whereas in relation to the medical treatment of her condition by the RCC staff, she relied on the professionals. The confirmatory interviews made it more evident that the degree to which one wishes to be actively involved depends on personal preferences. While some preferred to deliver data, but did not pay additional attention to it, others took an interest in following along themselves.

### Well-being

When participating in either of the services most informants experienced better physical, mental, and social health, which contributed to a positive change in well-being.

Informants suggested that exercise accomplished on a fixed weekly basis by a facilitator and together with peers creates motivation to be active. Those enrolled in the virtual RCC service were encouraged to acquire exercise equipment. Those who had more understanding of equipment and data monitoring, as well as users who are skilled and receptive to using technology, seemed to be more likely to own and use an at-home exercise bike:

The doctor (from the virtual RCC) said that it would be a good idea to buy an exercise bike, and he is completely right, so we have done that and we are using it

ID D3

Even though virtual services can motivate to acquire exercise equipment, informants from the confirmatory interview emphasized that physical presence and having exercise as a group activity is a superior method for increasing activity level.

You can do the exercises at home, but several agree that they find it difficult to be motivated when you are sitting home by yourself. So it’s kind of like motivation that you have to show up twice a week, you know?

ID I10

The rehabilitation program is the most motivating by far

ID C12

The social aspect of having a network of peers is also beneficial, and because many experience a decline in motivation, as well as social distance after the rehabilitation program ends, several reattended or wanted to reattend to maintain the achieved benefits.

I would like to re-attend. It’s a shame it’s only those 12 weeks. It makes you maintain... you know, it’s a little easier when you have it planned to make sure you actually go (exercise and socialize)

ID 15

The fact that the program is not a persistent offer might reduce the positive impact on well-being. This calls for a more sustainable service.

During the confirmatory interviews, when asked about whether the group-based activity could be carried out in a virtual format, the importance of the social aspect was highlighted, creating different opinions about the subject. While some appreciated the idea as this would exclude obstacles related to transportation, create relationships across distances, and make it easier to follow through during the COVID-19 pandemic, others argued that the social aspect cannot be replaced virtually and that the motivation for exercise declines in an at-home setting.

Well, there isn’t a lot of socializing in that kind of a digital course

ID C13

I have thought about it. On one hand, it would be optimal that you wouldn’t have to go places now with the high amount of infected (from Covid-19) (...) on the other hand, it’s nice to get that push to go out and be physically around other people (...) it’s also really good for people who are lonely

ID C12

Being monitored, having medicine for acute treatment available, and having close contact with well-known RCC staff can improve well-being by making the individual feel safe and secure. The professional network can benefit social interaction related to care and medical decisions in a different, but valuable way that sets them apart from that of peers. These components are, in this case, enabled by a virtual service. Those who have experienced both the rehabilitation program and the virtual RCC service describe the services as being very distinct:

It’s completely different services one would say, right? And in reality, services to different stages of the disease. One should attend the rehabilitation program earlier before it was necessary to be bombed with medication. In the rehabilitation program, there is the good aspect of the social, one could say. That’s not the case for the telemedicine (virtual RCC service). But with the telemedicine (...) you could be pretty sure to be monitored by a professional, and that’s pretty safe and secure.

ID C12

While the rehabilitation program supports physical activity, social interaction, and teaching coping mechanisms, the virtual RCC offers close contact with COPD specialists, the ability to follow their condition through monitoring, and acting on exacerbations through available medication.

## Discussion

### Principal Findings

For people living with COPD, contact with HCPs is a key aspect of care, both medically and emotionally. It is important to have close contact with HCPs and feel understood, properly medicated, and motivated, all of which can have a significant impact on their well-being. There is a desire to experience coherent care where HCPs are specialized in the disease, familiar, and in close contact. This can be supported by virtual services in which continuous monitoring, at-home medicine for acute treatment of exacerbations, and quick access to a HCP who knows the individual can promote self-management and provide a feeling of security. It can also provide the individual with a deeper understanding of their condition, improve medication adherence, and in some cases prevent hospitalizations.

Delivering information in a relevant and tailored manner is important for the receptiveness of the information. Many informants also suggest that having regular in-person meetings with a network of peers and professionals can promote knowledge about how to cope with the condition, which can motivate them to make health-related decisions. Motivation to exercise is also reinforced when performed as a collective activity with peers as this can create a sense of camaraderie and socialization among peers. Although encouragement by the RCC staff to invest and use at-home exercise equipment is effective in increasing activity levels for some, being physically active together with peers supervised by a professional is highlighted as the preferred way of performing and increasing physical activity.

When given the opportunity, some individuals prefer to take advantage of professional and peer networks for therapeutic social support. This is due to these networks having more insight and knowledge of people living with COPD and because some are worried about “burdening” their relatives and network.

The identified potential barriers to the benefits of the rehabilitation programs are related to the limited period in which the rehabilitation service was offered (5-12 weeks). Many experienced a decline in motivation to exercise and keep in contact with peers after the program has ended, and therefore expressed a wish to reattend a rehabilitation program to maintain benefits. Technology can facilitate monitoring and utilize reported data to support tailored treatment, but technology itself is of less significance compared with factors such as support and close contact with HCPs. It can even act as a barrier to optimal utilization of the service due to different levels of skills and preferences of the participants. The identified potential barriers to utilizing the benefits of the virtual RCC service are related to differences in levels of contact depending on disease severity and skills related to the practical use of equipment. Having a less severe state of COPD means having less contact, and thereby an experience of less social support by the RCC staff. Nevertheless, the wish for more contact was expressed with hesitance by those who did not often experience exacerbations, presumably because the participants overall felt gratitude toward the service and did not wish to appear unappreciative. The potential of social support might not be fully utilized in the 2 services included in this study as a result of these barriers.

Another barrier is related to the use of digital equipment. Although the sharing of personal data did not appear to worry the individuals, having difficulty in using the equipment or lacking the mental surplus to use it regularly can cause individuals to abandon the equipment, the RCC service altogether, or not fully utilize the potential of use, thus missing the benefits. This creates a barrier that makes the individual’s skills in technology a contributing factor to the success of engaging in a virtual service. Therefore, when considering digital solutions, it is important to consider the individual’s skill, preferences, and experience, as well as enabling alternative options to technology-based monitoring to preserve close and collaborative contact with HCPs. Further exploration is needed to assess how to accommodate the identified barriers related to the use of technology.

### Comparison With Prior Work

The findings suggest that close contact with HCPs and the use of a virtual service with digital equipment can make the individual feel more secure. It also has the potential to promote self-management as individuals can gain an understanding of their condition and treatment and obtain tools to actively engage in their treatment with solicited advice from HCPs. This is in line with the discoveries made by Nissen and Lindhardt [43] who in their Danish study found that patients with COPD who used a telehealth solution similar to that in this study felt more secure and achieved a higher understanding and competence in self-management in relation to their disease. According to a systematic review by Gorst et al [44], some of the benefits of receiving telehealth are improved self-care, increased access to health care, improved health knowledge, ease of use, peace of mind, convenience, effective health management, appreciation of telehealth nurses, and believing telehealth to be as good or better than in-person care.

In a previous study [21] exploring the effect of telemedicine on patient-reported outcomes for those with COPD, participants reported feeling less active in managing their health after inclusion. Our findings may explain this as our informants experienced that the access to the tools and support was easier, and that the feeling of safety related to being monitored by the RCC staff eases their burden, thereby feeling less active in managing their own health.

Although we and others have found several benefits of telehealth, the use of technology may still impose a barrier for some users. This aligns with the findings by Gorst et al [44] who reported that barriers to using telehealth and reasons for declining to receive the service were, among others, related to technical problems. According to Gorst et al [44], other barriers were related to a preference for in-person care. Interestingly, but also contradictorily, they found that reported facilitators for telehealth believed that telehealth is as good as or even better than in-person care. Either way, preferences related to personal contact appear to be a factor in one’s attitude or experience toward receiving telehealth. The informants in our study did not express feeling compromised with regard to contact with HCPs because of the service. Instead, the technology seems to support close and quick contact with HCPs. However, this is dependent on the condition of their COPD, where those with fewer exacerbations have less contact with RCC staff and expressed a desire to be checked upon more often. The importance of medical and social support from HCPs perhaps explains why the attention to technology is of less significance for our informants unless they encounter problems in the daily use of these technologies. It might be perceived as a tool more than an individualized care component.

Our findings that group-based physical activity motivates and increases activity levels and that a network of peers provides knowledge and socialization correlate with findings from other studies [13,45,46]. These studies found that individuals living with COPD benefited on a social and psychological level from participating in group-based exercise interventions with coaching. They concluded that camaraderie and motivation are created when exercising with others with a similar condition and that depression and anxiety related to breathlessness decrease upon attending group activities. These studies also found that while many own exercise equipment at home, they do not use them due to a lack of motivation, and concluded, similar to this study, that participants wished to continue attending their programs to maintain benefits [45].

Interestingly, this subject was not discussed by participants of the virtual service, but could be explained by the service already being continuous, based on findings by Emme et al [47] who found that the benefits of telehealth depend on the availability and use of equipment. This means that the achieved coping in disease handling could not be sustained after cessation of the daily monitoring including virtual ward rounds. This points to a general tendency to wish for more sustainable services, which is in contrast to the GOLD recommendations for the duration of rehabilitation programs (ie, 6-8 weeks) [2].

A previous study [13] addressing cancer survivors and physical activity found a connection between the level of technology readiness and physical activity preferences. People scoring low in technology readiness generally tend to prefer a social or coaching approach, whereas those who score high in technology readiness generally prefer individual physical activity (eg, fitness centers and apps). There is a correlation between socioeconomic status and technology readiness, with lower socioeconomic status being associated with lower technology readiness and a preference for performing physical activity in a social context [13,48]. Our data support these findings as it seemed that those who were more engaged with their digital equipment were more likely to own and use an at-home exercise bike compared with those less engaged with their digital equipment. Yet, the general tendency was that for those who had experienced both services, there was a preference for group-based physical activity with coaching, which is similar to the findings in cancer survivors, where those with lower socioeconomic status preferred group-based activity. It also aligns with the GOLD recommendations that supervision in exercise interventions is important for its effectiveness in individuals living with COPD [2].

As the virtual service and the rehabilitation program are described as 2 distinct types of services with different benefits, we suggest that a combination of the 2, based on the individuals’ preferences and skills, can improve future care for patients living with COPD. Both literature and our study suggest that a lasting offer of these services is the most beneficial as motivation, physical activity achieved, and other lifestyle changes decline after the service ends.

Whether physical activity with a network of peers could also be successfully carried out in a virtual format is yet to be fully discovered. The informants in this study had different preferences for virtual training with peers, which calls for a more systematic investigation of this area in the future. Future research should also be carried out to assess how to accommodate barriers related to technology.

### Limitations

By recruiting from the TEMOCAP study, there might have been a narrow availability of informants with different backgrounds. There is a chance that those who accepted the invitation to the TEMOCAP study are more open to new ways in which health care can be provided. This could result in our informants having fewer reservations toward the use of digital technologies in treatment compared with the general population. This could explain why none of our informants reported having reservations toward the use of technology in their treatment in general, except when experiencing technical difficulties. Males were more likely to accept participation in the study compared with females, which should be considered when interpreting the study results. This ratio is different from that of the COPD population and may add to a positive opinion around technology use and a lack of other perspectives that may have been introduced if more females had participated. We had attempted to mitigate this by inviting an equal number of females and males for the second round of interviews. Further, it may have been difficult to interpret nonspoken signals (eg, body language such as gestures and restlessness) as the interviews were conducted virtually due to COVID-19. The use of a screen may also reduce the possibility to establish a relationship with the informants, and thereby a risk of them not disclosing more sensitive matters.

The limited knowledge about how the informants feel about the external use of their data, and whether this could be a barrier for some, could be due to a lack of focus on this topic in our interview guide. The topic was only briefly mentioned by a couple of informants, which only indicates their experience.

### Strengths

To have a better understanding of our findings and strengthen the information power, data from the interviews were triangulated with a second round of interviews by adding the informants’ perspective of the combined experience of the services mentioned. With our narrow focus of interest, which according to Malterud et al [49] increases the likelihood of information power, the results might not be representative on a wider scale, but seem to be representative for a population recruited in this way.

As author KP had a possible conflicts of interest, he did not participate in the interview process or analysis, but participated only in the planning and discussion processes. TK and EHJ are external to Epital Health and the context of virtual services, but prior to the study had obtained knowledge about telemedicine services and people living with chronic conditions in a Danish setting.

### Conclusions

The findings call for a future design to address medical as well as mental care, individual preferences, the potential of peers and facilitators, and different levels of skills to overcome potential barriers of technology. Taking preferences and skills into account, this study points to a combination of the identified valued elements from virtual and in-person services as a foundation for the future care of individuals with COPD to provide lasting medical and social support across allied HCPs and peers. Lasting offers of these services are recommended to maintain motivation and achieved lifestyle changes.

## Appendix

Multimedia Appendix 1 Interview guide: first interview.

Multimedia Appendix 2 Interview guide: confirmatory interview.

## Abbreviations

BDI: The Beck Depression Inventory
COPD: chronic obstructive pulmonary disease
COREQ: Consolidated Criteria for Reporting Qualitative Research
ECM: Epital Care Model
GOLD: Global Initiative for Chronic Obstructive Lung Disease
GP: general practitioner
HCP: health care professional
HeiQ: Health Education Impact Questionnaire
RCC: response and coordination center
READHY: Readiness and Enablement Index for Health Technology
WHO: World Health Organization
WHODAS II: World Health Organization Disability Assessment Schedule 2.0

## Data Availability

Our institution follows the FAIR (Findability, Accessibility, Interoperability, and Reusability) principles. We therefore in principle always want to make our data accessible but as this study addresses interview data from a small population, we do not find it possible to make these data available without disclosing recognizable health and personal data. The data set is therefore not available. Please contact us if you need any specific information.
