# Supplementary material for: Experiences With In-Person and Virtual Health Care Services for People With Chronic Obstructive Pulmonary Disease: Qualitative Study
Source: JMIR Rehabil Assist Technol. 2023 Aug 14;10:e43237. doi: 10.2196/43237 (PMC10463085; doi:10.2196/43237)
Supplement: Multimedia Appendix 1 [file rehab_v10i1e43237_app1.pdf]

Interview guide

Introduction

Thank you for participating in this interview. I want to start by telling a little about myself. My name is Emma/Thea and I study Health and Informatics at the University of Copenhagen. In collaboration with a fellow student, I am writing a bachelor's project which focuses on the well-being and treatment offered to individuals with COPD. Therefore, I would like to hear a little about how you experience your treatment and how you feel.
The interview will last approx. an hour and there are no wrong answers to the questions I ask you.
We record the interview to be able to go back and hear and analyze some of the things we talked about. You can withdraw your consent to participate at any time and this does not affect anything in relation to your treatment.

To begin with, I would like to ask you a few questions about yourself:

How old are you?
What education do you have?
What is your marital status?
How long have you lived with COPD?

Semi-structured interview

Themes interest	Themes/Dynamic Questions	Follow-up questions
How do the individuals maintain their treatment and how do they manage their condition?	Would you like to tell me a bit about what kind of treatment you receive for your COPD?<br><br>What does a typical day look like for you when you receive treatment?	Do you have any regular routines?<br><br>Do you go to check-ups?<br><br>What medication are you receiving?

	How much time do you spend on an everyday basis on treatment for your COPD?	Is there anyone helping you with your treatment or medication?<br><br>Do you use any appliances or tools? How do they work?<br><br>Did you get them in connection with your treatment or did you buy them yourself?<br><br>What is the time spent on?<br><br>Are some things more time-consuming than others?
What influence does the treatment/service have on the individuals' well-being and how do they handle exacerbations?	How does living with COPD affect you in your everyday life?<br><br>Do you sometimes experience a worsening of your condition?<br><br>Can you tell me a little about how the worsenings manifest themselves?<br><br>What do you do when experiencing severe shortness of breath?	Do you have any examples of how it affects you in your everyday life?<br><br>How much do you think about your COPD in your everyday life?<br><br>Who do you talk with about your COPD?<br><br>How do you deal with these exacerbations when you feel them?<br><br>Do you seek help when you experience a worsening?<br><br>How do you know if the situation is something that calls for you to seek help?<br><br>Where do you seek help? Family/friends/doctor?
Well-being: WHO-5 well-being index. How do the	Looking back over the past two weeks, can you say a few words about how you've been?	

citizens in the two groups thrive?	Have you felt that you have been able to take it easy and relax?<br><br>How has your energy and activity level been in your daily life?<br><br>Have you felt well-rested in the morning when you wake up?<br><br>Have you done anything that interests you in your everyday life?	
Are there any disadvantages to the form of treatment that the two groups receive and how does it affect the individuals' satisfaction with their treatment?	Are there certain days when you have had treatment/check-ups/monitored yourself that you remember particularly clearly because of a certain experience? It can be both positive or negative.<br><br>Is there anything about your treatment that you find difficult or easy?<br>What could it be?<br><br>Is there something you, in your opinion, are missing in your treatment?	Try to think about that day. What did you experience?
Debriefing and round up	Thanks. These were the questions I had prepared for today. Is there anything you would like to say that you think is important, but that we haven't talked about?<br><br>Do you have any questions for me?<br><br>Thank you for that and thank you for your time. If any questions should arise, you are very welcome to contact me.	
