# Supplementary material for: Experiences With In-Person and Virtual Health Care Services for People With Chronic Obstructive Pulmonary Disease: Qualitative Study
Source: JMIR Rehabil Assist Technol. 2023 Aug 14;10:e43237. doi: 10.2196/43237 (PMC10463085; doi:10.2196/43237)
Supplement: Multimedia Appendix 2 [file rehab_v10i1e43237_app2.pdf]

Interviewguide

The purpose of this interview is to talk about the similarities and differences that you have experienced by participating in a COPD rehabilitation program and in the telemedicine service offered by *the Epital* (Epital Health). You have experienced both and therefore I would like to hear about what you think was particularly good or less good about one service versus the other.

I am neither affiliated with the Epital nor connected to those who offer the COPD course, so I am completely neutral and there are no wrong answers in this interview. I would like to emphasize that you can withdraw your consent to participate at any time.

Remember to ask if they are OK with the possibility of going over time

Questions for demographics:

Age, number of years lived with COPD, marital status, and education level.

Part 1: My interview will be divided into two parts. Here in the first part, I would like to hear a little about what the two services have consisted of for you. Next, I would like to talk about the differences between the two services in relation to what has been good and what has been difficult.

Question 1:

Can you tell me about what the COPD course you attended involved? What did a typical day look like for you when you were on this course and how often did you go?

Question 2:

Can you tell me about what the telemedicine service entails/entailed? As well as what a typical day looks like when you use this service.

Question 3:

If we are to compare these two types of service:

Were there any good things about the COPD rehabilitation service that you think the telemedicine service lacked? You don't have to answer right away, you can take a minute to think about it.

Were there any good things about the telemedicine service that you think the COPD rehabilitation service lacked?

Question 4

Were there any disadvantages to the COPD rehabilitation service? Something you think didn't work or could be better?

Likewise, are there any disadvantages to the telemedicine service from the Epital? Something you think didn’t work or could be better?

Part 2:

Here in the second part of the interview, I would like to present you with some findings from previous interviews. I would like to introduce them to you and ask if you agree with them or not. It is important for me to mention that it is both interesting and relevant to hear what you cannot recognize as well as what you may well recognize. So there are no wrong answers.

Question 5

Some of those I have previously spoken to said that the COPD rehabilitation service gave them a network, both in the form of some health care professionals as well as other people who also had COPD, and that the network provided a place where one could share experiences, give each other good advice and support each other. Is it something you recognize?

What are your thoughts on that about the Epital? Is there also a network there?

Question 6

Some reported that they had a different experience with their COPD treatment from the general practitioner to the telemedicine service. Is this something you recognize?

Follow-up: Some mentioned something with medication, time, and commitment

Question 7

What are your thoughts on whether this COPD rehabilitation service could have been carried out digitally, i.e., sitting in front of your computer or phone, seeing each other and receiving the same information, etc.?

Do you think that would have made the experience different?

Can you try to put some words into how it would have been different?

Question 8

Some said that it gave them a sense of security to know that they had some acute medicine in the home, which they could take when they experienced exacerbations, and that they could be quickly in contact with health care personnel. Is this something you recognize?

**Question 9**<br><br>Some said that via the Epital they had gained more insight into their condition by being able to monitor and follow their data. Is it something you recognize?<br><br>What about the COPD rehabilitation service: Has the course affected your habits or well-being? E.g., how you eat, whether you smoke, exercise?
**Question 10**<br><br>Some said that the fact that there are some healthcare professionals who continuously monitor their submitted data helped to provide a sense of security because they felt that someone was 'watching' them. Is it something you recognize?
**Question 11**<br><br>Some said that they generally think that these services made them more active. For the COPD rehabilitation service, it was because the exercise was integrated into the course, and for the telemedicine service because they are encouraged to do so. Is this something you recognize?
**Question 12**<br><br>During the previous interviews, it became clear that this aspect of IT or technology was mentioned to a lesser extent compared to elements such as support and the efforts of the healthcare staff. It didn't seem like the use of technology itself was something the participants thought much about in everyday life, except when it didn't work. Is this something you recognize?
